# Experimental and Theoretical Study of N_2_ Adsorption on Hydrogenated Y_2_C_4_H^−^ and Dehydrogenated Y_2_C_4_^−^ Cluster Anions at Room Temperature

**DOI:** 10.3390/ijms23136976

**Published:** 2022-06-23

**Authors:** Min Gao, Yong-Qi Ding, Jia-Bi Ma

**Affiliations:** Key Laboratory of Cluster Science of Ministry of Education, Beijing Key Laboratory of Photoelectronic/Electrophotonic Conversion Materials, School of Chemistry and Chemical Engineering, Beijing Institute of Technology, Beijing 102488, China; 3120201255@bit.edu.cn (M.G.); 3120195628@bit.edu.cn (Y.-Q.D.)

**Keywords:** N_2_ adsorption, mass spectrometry, density functional theory calculations

## Abstract

The adsorption of atmospheric dinitrogen (N_2_) on transition metal sites is an important topic in chemistry, which is regarded as the prerequisite for the activation of robust N≡N bonds in biological and industrial fields. Metal hydride bonds play an important part in the adsorption of N_2_, while the role of hydrogen has not been comprehensively studied. Herein, we report the N_2_ adsorption on the well-defined Y_2_C_4_H_0,1_^−^ cluster anions under mild conditions by using mass spectrometry and density functional theory calculations. The mass spectrometry results reveal that the reactivity of N_2_ adsorption on Y_2_C_4_H^−^ is 50 times higher than that on Y_2_C_4_^−^ clusters. Further analysis reveals the important role of the H atom: (1) the presence of the H atom modifies the charge distribution of the Y_2_C_4_H^−^ anion; (2) the approach of N_2_ to Y_2_C_4_H^−^ is more favorable kinetically compared to that to Y_2_C_4_^−^; and (3) a natural charge analysis shows that two Y atoms and one Y atom are the major electron donors in the Y_2_C_4_^−^ and Y_2_C_4_H^−^ anion clusters, respectively. This work provides new clues to the rational design of TM-based catalysts by efficiently doping hydrogen atoms to modulate the reactivity towards N_2_.

## 1. Introduction

More than 99% of the global nitrogen exists in the shape of gaseous dinitrogen (N_2_) in the atmosphere, yet most organisms can only metabolize nitrogen-containing substances such as NH_3_ rather than N_2_ directly. Although N_2_ is the main nitrogen source for most natural and artificial nitrogen-containing compounds, the high bond dissociation energy (9.75 eV) and the large HOMO–LUMO gap (10.8 eV) render its adsorption and activation an enormous challenge in chemistry [1,2,3,4]. Scientists regularly rely on transition metal (TM) centers to catalyze the nitrogen conversion processes [5,6,7]. The initial and critical step in the complicated reduction of dinitrogen is the adsorption of N_2_ molecules at the TM center [8,9]. The fixation of nitrogen in industry is carried out at metal-based (Fe^−^ or Ru^−^) catalysts under extremely high temperatures (300–500 °C) and high pressures (100–300 atm), involving the disadvantages of large energy consumption and greenhouse gas emission [10,11,12]. Thus, it is vital to develop mild, energy-saving, and environment−friendly catalytic systems for N_2_ fixation at ambient conditions. The activation of nitrogen by transition metal compounds with the involvement of hydrogen atoms is of particular interest, while the most common feature of N_2_ hydrogenative cleavage is the participation of metal hydride bonds [13,14,15]. A literature survey [13] shows that metal hydride bonds have several important roles: (1) as a hydrogen source; (2) as an electron source for N_2_ reduction; (3) as a powerful reducing agent for the removal of activated nitrogen atoms; and so on.

As an ideal model of condensed-phase systems, gas-phase clusters can study chemical reactions and reveal related mechanisms at the strictly molecular level by simulating active sites. [16,17,18,19]. Several theoretical and experimental studies have reported the reactivity of metal species with nitrogen, however, only a few metal species such as, Sc_2_ [20], Ta_2_^+^ [21], V_3_C_4_^−^ [22], Ta_2_C_4_^−^ [23], NbH_2_^−^ [24], Ta_3_N_3_H_0,1_^−^ [25], Sc_3_NH_2_^+^ [26], FeTaC_2_^−^ [27], and AuNbBO^−^ [28] have been characterized to cleave the N≡N triple bond completely. It can be seen that for the studies on N_2_ adsorption in the gas phase, there are few metal species, and they mainly focus on the early transition metals. In the previous work, we found that a suitable number of hydrogen atoms has an influence on the reactivity of transition metal-containing clusters with N_2_ [24,25,26,29,30]. Sc_3_NH_2_^+^ [26] can effectively realize the activation of N_2_ by H_2_, which is based on the regulation of N_2_ reduction by two H atoms. Ta_3_N_3_H_0.1_^−^ is an example that highlights the importance of the assisted reactivity of a single hydrogen atom, and the reactivity of Ta_3_N_3_H^−^ is higher by a factor of five compared with that of Ta_3_N_3_^−^ due to the hydrogen atom changing the charge distribution and geometry [25]. How can hydrogen atoms be efficiently doped to modulate the reactivity of TM-containing systems towards N_2_ at the molecular scale? Considering the previous exploration of the Sc systems and the fact that Sc and Y belong to the same group, Y_2_C_4_^−^ and Y_2_C_4_H^−^ cluster anions were synthesized, and the reactivity towards N_2_ was investigated by mass spectrometry and DFT calculations, to answer this question. This work clearly revealed that Y_2_C_4_H_0,1_^−^ anions can adsorb N_2_, and the hydrogen atom greatly enhances the reactivity of Y_2_C_4_H^−^ towards N_2_.

## 2. Results and Discussion

**Figure 1:**
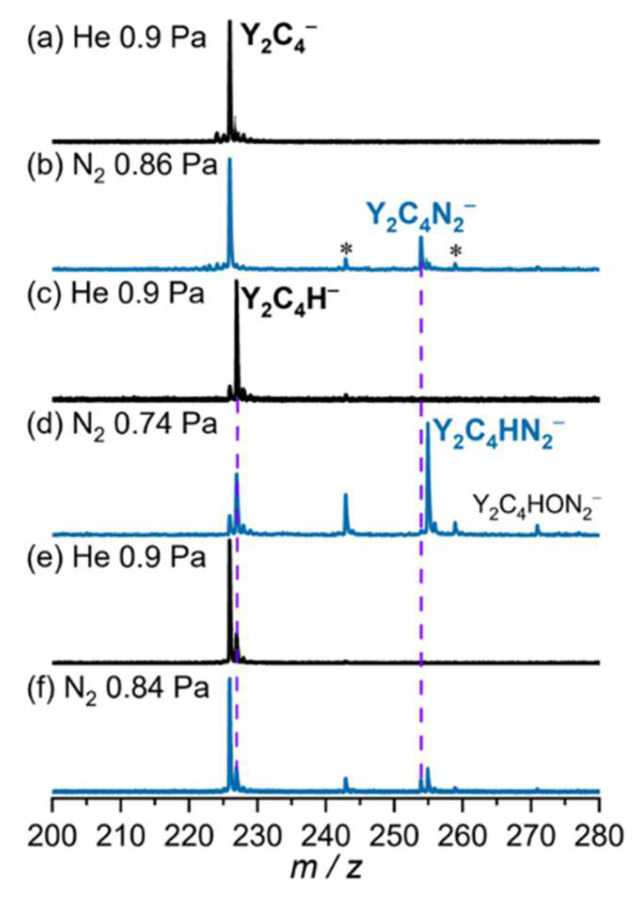
TOF mass spectra for the reactions of (**a**) mass-selected Y_2_C_4_^−^ with He and (**b**) N_2_ for 6 ms, (**c**) mass-selected Y_2_C_4_H^−^ with He and (**d**) N_2_ for 14 ms, and (**e**) the coexisting Y_2_C_4_^−^ and Y_2_C_4_H^−^ clusters with (**f**) N_2_ for 10 ms, respectively. The effective reactant gas pressures are shown. The asterisked peaks (*) are Y_2_C_4_OH^−^ and Y_2_C_4_O_2_H^−^, due to the reactions with residual water in the LIT. Black bold, blue bold and black font represent reactants, products and impurities, respectively.

The time-of-flight (TOF) mass spectra of laser ablation-generated, further mass-elected Y_2_C_4_^−^ and Y_2_C_4_H^−^ cluster anions reacting with N_2_ under thermal collision conditions in a linear ion trap (LIT) reactor are shown in Figure 1. The mass spectra for the generation of Y_2_C_4_H_0,1_^−^ clusters has been given (Supplementary Figure S1). Upon the interactions of Y_2_C_4_^−^ and Y_2_C_4_H^−^ with N_2_, two adsorbed complexes that are assigned as Y_2_C_4_N_2_^−^ and Y_2_C_4_HN_2_^−^ are observed (Figure 1b,d), suggesting the following channels in Equations (1) and (2):

Y_2_C_4_^−^ + N_2_ → Y_2_C_4_N_2_^−^ (1)

Y_2_C_4_H^−^ + N_2_ → Y_2_C_4_HN_2_^−^ (2)

Compared with Y_2_C_4_^−^, Y_2_C_4_H^−^ shows a higher reactivity towards N_2_ under the same reaction conditions in Figure 1f. Besides the major products, two weak peaks in Figure 1 are assigned to Y_2_C_4_OH^−^ and Y_2_C_4_O_2_H^−^, generated from the reaction of Y_2_C_4_H_0,1_^−^ anions with water impurities in the LIT. The pseudo-first-order rate constants (*k*_1_) for the reactions one and two are estimated to be (3.7 ± 0.8) × 10^−12^ cm^3^ molecule^−1^ s^−1^ and (6.2 ± 1.3) × 10^−14^ cm^3^ molecule^−1^ s^−1^, which are based on a least-square fitting procedure, corresponding to reaction efficiencies (*Φ*) [31,32] of 0.6% and 0.01%, respectively. Additionally, the signal dependence of product Y_2_C_4_H_0,1_N_2_^−^ ions on N_2_ pressures was obtained, which are derived and fitted with the mass spectrometry experimental data (Supplementary Figure S2).

BPW91 calculations are performed to investigate the structures of reactant Y_2_C_4_H_0,1_^−^ anion clusters (Supplementary Figure S3), as well as the reaction mechanisms between Y_2_C_4_H_0,1_^−^ and N_2_. The lowest-energy isomer of Y_2_C_4_^−^ (doublet, ^2^**IA1**, Supplementary Figure S3), which is 0.08 eV lower than its quartet isomer, is a *C*_s_−symmetric six−membered ring, with the Y-Y bond as the symmetry axis and two *C*_2_ ligands bonded to the two Y atoms. Moreover, the most stable isomer of Y_2_C_4_H^−^ (^1^**IA2**) has a hydrogen atom binding to the Y1 atom in the six-membered ring, similar to the Y_2_C_4_^−^ (^2^**IA1**), and it is 0.07 eV lower than the triplet state in energy (Supplementary Figure S3). Since the energies of the isomers are very close, their reaction paths are calculated. The results show that, in the reaction coordinates, the energies of the doublet and singlet stationary points and the products in the Y_2_C_4_^−^/N_2_ and Y_2_C_4_H^−^/N_2_ systems are lower than those of the corresponding quartet and triplet analogues, respectively (Supplementary Figure S4). Enthalpy and Gibbs free energies along with electronic and zero-point correction energies are added (Supplementary Table S1). The concentration of dinitrogen adducts in the gas phase is relatively low, so it is difficult to collect and continue to measure Raman spectra. Currently, it is difficult to characterize structures due to technical and instrumental limitations. Infrared multiple photon dissociation may be applied to reveal such types of anions. We have added the calculated infrared spectra (Supplementary Figure S5), and the vibrational frequencies may be used for future experimental identification of these clusters.

**Figure 2:**
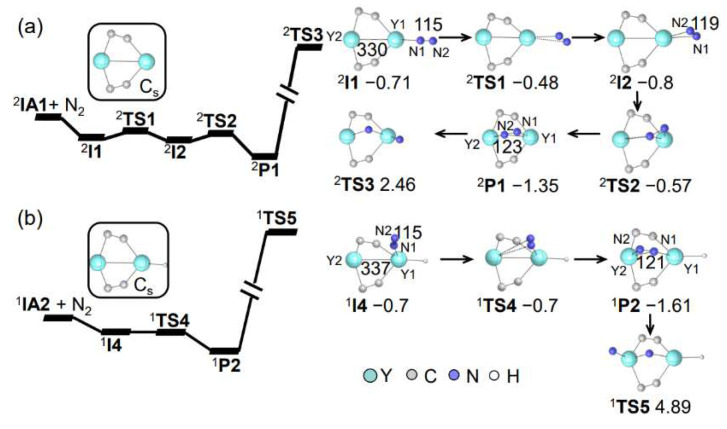
BPW91-D3-calculated potential energy surfaces for the reactions of Y_2_C_4_^−^ (**a**) and Y_2_C_4_H^−^ (**b**) with N_2_. The zero-point vibration-corrected energies (Δ*H*_0K_ in eV) of the reaction intermediates (**I1**–**I4**), transition states (**TS1**–**TS4**), and products (**P1**, **P2**), with respect to the separated reactants, are given. The bond lengths are given in pm. The green, blue, grey and white atoms represent Y, N, C and H atoms, respectively. Spin multiplicity is located in superscript.

The potential energy surfaces (PESs) of the most favorable reaction pathways are given in Figure 2. The N_2_ molecule is initially captured by the Y1 atom in both Y_2_C_4_^−^ and Y_2_C_4_H^−^ to form the end-on-coordinated complexes ^2^**I1** and ^1^**I4**. Notably, ^2^**I1** (−0.71 eV) in Figure 2a is as stable as ^1^**I4** (−0.70 eV) in Figure 2b, suggesting that the N_2_−adsorbed intermediates ^2^**I1** and ^1^**I4** are not the final products in the Y_2_C_4_^–^/N_2_ and Y_2_C_4_H^–^/N_2_ systems. As for the Y_2_C_4_^−^/N_2_ system, the coordination mode of N_2_ is further changed from *η*^1^ in ^2^**I1** to *η*^2^ in ^2^**I2** via ^2^**TS1**. During this process, the N-N bond length is elongated from 110 pm in free N_2_ to 119 pm in ^2^**I2**. Subsequently, the adsorbed N_2_ unit is anchored by two Y atoms via ^2^**TS2**, forming a Y-N-N-Y bridge; at the same time, a longer N-N bond of 123 pm is generated in ^2^**P1**. Note that the rupture of the N-N bonds encounters a high energy barrier (^2^**TS3**, +2.46 eV with respect to the separated reactants), so that further activation of N_2_ is hampered in this system.

**Figure 3:**
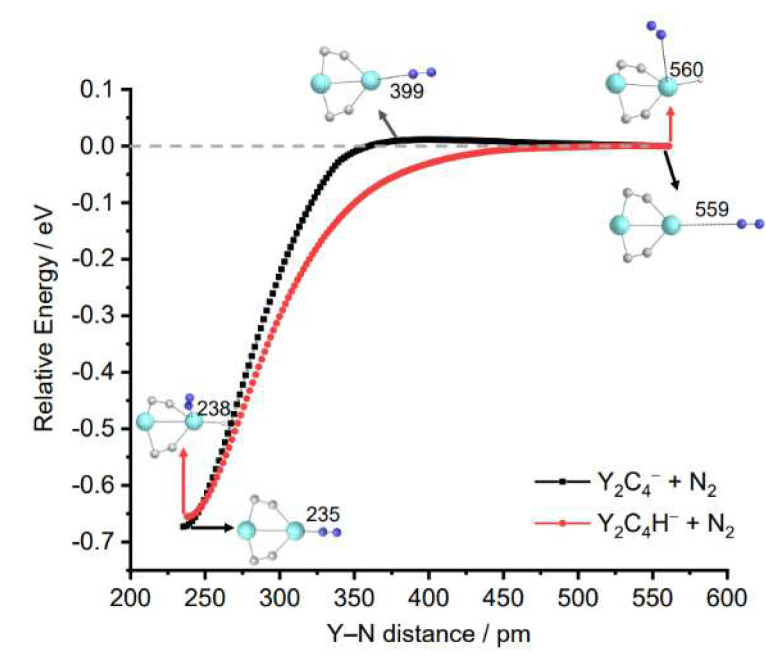
The BPW91-calculated relaxed potential energy curves of N_2_ approaching Y_2_C_4_^−^ and Y_2_C_4_H^−^ anions.

The reaction of Y_2_C_4_H^−^/N_2_ (Figure 2b) follows the similar mechanism. The complex is coordinated laterally to form a Y-N-N-Y bridge like ^2^**P1** by overcoming a negligible barrier ^1^**TS4**, and the activation energy (Δ*E*_a_, i.e., the energy difference between the encounter complex and the transition state) is lower than that of ^2^**I2** → ^2^**TS2** (Δ*E*_a_ = 0.23 eV) in Y_2_C_4_^−^. In the step of ^1^**I4** → ^1^**P2**, an elongation of the N–N bond from 115 to 121 pm occurs. Further cleavage of N–N is also hindered due to the positive energy barrier of 4.89 eV (^1^**TS5**). In addition, another adsorption of N_2_ on the Y2 atom (Supplementary Figure S6) that is not bonded with the hydrogen atom can be eventually trapped in ^1^**P2** by generating the *η*^2^-mode intermediate ^1^**I7**. In conclusion, the reactions of Y_2_C_4_H^−^ and Y_2_C_4_^−^ with N_2_ result in the formation of bridging adsorption products ^2^**P1** and ^1^**P2**, and the adsorbed N_2_ molecules are in the *η*^1^:*η*^2^ mode. As shown in Figure 3, the potential energy curves reveal that the adsorption process of Y_2_C_4_H^−^/N_2_ is more favorable kinetically compared to that of Y_2_C_4_^−^/N_2_, since it is barrier−free for Y_2_C_4_H^−^/N_2_. A small barrier exists in the shallow entrance channels when N_2_ approaches Y_2_C_4_^−^, which further explains the experimental observed low reaction rate constant for the dehydrogenated Y_2_C_4_^−^/N_2_.

**Figure 4:**
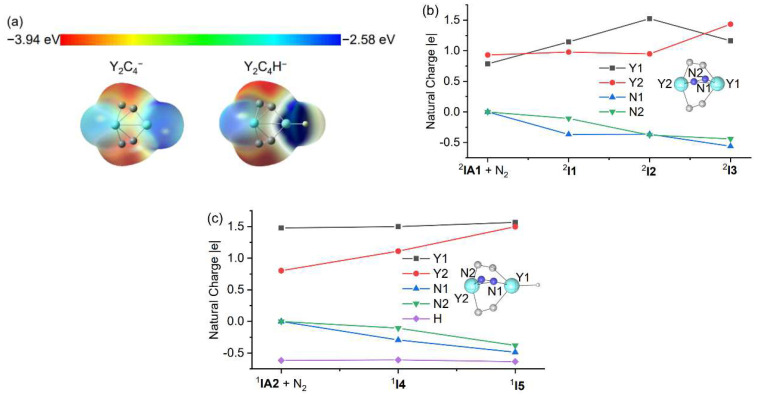
(**a**) Electrostatic potentials of the Y_2_C_4_H_0,1_^−^. Charges on atoms of stationary points along reaction coordinates of N_2_ absorption on (**b**) Y_2_C_4_^−^ and (**c**) Y_2_C_4_H^−^ clusters.

Frontier orbital analysis shows that the immobilization of the N_2_ ligand, as well as the formation of ^2^**P1** and ^1^**P2**, involve *d*-electrons transfer from the single-occupied molecular orbital-1 (SOMO-1) of Y_2_C_4_^−^ and the HOMO orbital of Y_2_C_4_H^−^ to the antibonding π*-orbitals of N_2_ (Supplementary Figure S7). The presence of hydrogen atoms enhances the reactivity of the cluster cations toward N_2_ since it changes the charge distribution. As shown in Figure 4a, the Y1 linked to the hydrogen atom on the Y_2_C_4_H^−^ cluster has more negative charges compared to Y_2_C_4_^−^, and it promotes π-back-donation. Note that the energy differences between the transition states and the separated reactants, which is the apparent barrier (Δ*E*^‡^), matters in gas−phase studies. The apparent barrier for Y_2_C_4_H^−^/N_2_ (Δ*E*^‡^ = −0.70 eV) is lower than that of Y_2_C_4_^−^/N_2_ (Δ*E*^‡^ = −0.48 eV), and the energy of ^1^**P2** is lower than that of ^2^**P1** (−1.61 eV vs. −1.35 eV). According to the Rice-Ramsperger-Kassel-Marcus (RRKM) theory [33], the internal conversion rate of **I4** → **TS4** (8.49 × 10^11^ s^−1^) is 32 times larger than that of **I2** → **TS2** (2.65 × 10^10^ s^−1^). These theoretical results are consistent with the experiments.

To further improve the understanding of Y_2_C_4_H_0,1_^−^/N_2_ systems, NBO analysis along reaction coordinates was performed (Figure 4b,c). The charge details were added (Supplementary Table S2). In the adsorption processes **IA1** → **I1** and **IA2** → **I4** of Y_2_C_4_H_0,1_^−^/N_2_, the yttrium atoms transfer 0.37 *e* and 0.29 *e* to the N1 atom, respectively, leading to the formation of the Y-N1 bonds, while two N2 atoms in Y2C4^−^ and Y_2_C_4_H^−^ only increase by 0.11 *e*. In the subsequent steps **I2** → **P1** and **I4** → **P2** for the formation of the N2-Y2 bonds, more electrons are stored in the two nitrogen atoms, resulting in the gradual elongation of the N-N bonds. Overall, the electrons required for the N_2_ adsorption by the Y_2_C_4_^−^ and Y_2_C_4_H^−^ clusters are mainly provided by Y atoms with total transferred amounts of 0.88 *e* and 0.78 *e*, respectively. Differently, two and one Y atoms are the electron donors in Y_2_C_4_^−^ and Y_2_C_4_H^−^, respectively. The active Y1 atom in Y_2_C_4_^−^ (**IA1**) has more 5s electron occupancies (5s^1.10^ 4d^1.03^), which causes an unfavorable approach and a high σ-repulsion on the N_2_ molecule. When one hydrogen atom on the Y_2_C_4_^−^ (^2^**IA1**) cluster bonds to form Y_2_C_4_H^−^ (^1^**IA2**), the natural charge on the Y1 increases from 0.79 *e* to 1.48 *e*; at the same time, more 4*d* and less 5*s* electron occupancies are located (5s^0.38^ 4d^1.12^), which can make N_2_ more accessible to the Y_2_C_4_H^−^ cluster anions. The values of bond orders of Y-Y bond in Y_2_C_4_H_0,1_^−^ anions are an important indicator for the ability of storing electrons, which increases from 0.55 in Y_2_C_4_^−^ (^2^**IA1**) to 0.66 in Y_2_C_4_H^−^ (^1^**IA2**). Therefore, although hydrogen appears to be a bystander in N_2_ adsorption, its presence indeed stores more electrons in the Y-Y bond and facilitates N_2_ adsorption. It can be concluded that the hydrogen atom in the Y_2_C_4_H^−^ cluster significantly affects the charge distribution and electronic structure, and a suitable number of hydrogen atoms can enhance the reactivity towards N_2_.

## 3. Methods

### 3.1. Experimental Methods

The metal carbide clusters were generated by laser ablation metal target (made of pure yttrium powder) (Jiangxi Ketai New Materials Co. Ltd, Jiangxi, China) seeded at 2‰ CH_4_(Beijing Huatong Jingke Gas Chemical Co. Ltd, Beijing, China) in a helium carrier gas (backing pressure 4 atm). The pulsed laser is a 532 nm laser with 5–8 mJ/energy pulses and 10 Hz repetition rate (140 Baytech Drive, San Jose, CA, USA). Y_2_C_4_^−^ and Y_2_C_4_H^−^ anion clusters were mass-selected by a quadrupole mass filter (QMF) (China Academy of Engineering Physics, Mianyang, Sichuan, China) [34] and subsequently entered into a linear ion trap (LIT) reactor (homemade) [35]. After being confined and thermalized by the pulsed gas He for about 2 ms, they interacted with N_2_ for about 6 ms and 14 ms, at room temperature, respectively. The anion clusters were ejected from the LIT and then detected by a reflection time-of-flight mass spectrometer (TOF-MS) [36]. The rate constants of the reactions between Y_2_C_4_H_0,1_^−^ cluster anions and N_2_ were described [37]. A schematic diagram of the experimental apparatus is shown in ref [34]. 

### 3.2. Computational Methods

All DFT [38] calculations were formed using the Gaussian 09 [39] program package to explore the structures of reactant clusters Y_2_C_4_H_0,1_^−^ and the mechanistic details of Y_2_C_4_H_0,1_^−^ with N_2_. To give the best interpretation of the experimental data, we calculated the dissociation energies of the Y-Y Y-C, N-N and C-C (Supplementary Table S3) bonds using 20 methods. The results show that BPW91 functional [40,41,42] performs very well. For application of basis sets in reaction systems, the def2-TZVP [43] basis set was used for the Y atom, and the 6 − 311 + G * basis sets [44,45] were selected for the C, H, and N atoms, which were applied in other systems containing these elements [24,27,46]. The zero-point vibration corrected energies (Δ*H*_0K_ in eV) in unit of eV are reported. Vibrational frequency calculations must be performed for the geometric optimization of the reaction intermediates (**I**Ms) and transition states (**TS**s) [47]. Intrinsic reaction coordinate [48] calculations were employed to ensure whether each TS was connected to two appropriate local minima. DFT-D3 correction for the complexes were contained in the system. Natural population analysis was performed using NBO 6.0 [49], and the orbital composition was analyzed by the method of natural atomic orbitals employing the Multiwfn program [50].

## 4. Conclusions

In summary, the reactions of Y_2_C_4_H^−^ and dehydrogenated Y_2_C_4_^−^ cluster anions with N_2_ have been investigated experimentally and theoretically. The experimental results indicate that the reaction rate constant of Y_2_C_4_H^−^/N_2_ is higher by a factor of 50 compared with that of Y_2_C_4_^−^/N_2_. DFT calculations indicate that the differences are caused by the different charge distributions and the bonding of the additional hydrogen atom to the yttrium atom in the Y_2_C_4_H^−^ cluster, resulting in more 4d electron occupancies and thus more efficient π-back-donation bonding with N_2_ molecules. The electron donor atoms of Y_2_C_4_^−^ and Y_2_C_4_H^−^ anion clusters are different, for Y_2_C_4_^−^, two Y atoms donate electrons, while only one Y atom donates electrons in Y_2_C_4_H^−^. Storing more electrons in the Y-Y bond is also an important influence of the hydrogen atom on the reactivity of Y_2_C_4_H^−^ to N_2_. This study clearly reveals the significance of hydrogen-assisted reactions in N_2_ adsorption processes. Attaching an appropriate number of hydrogen atoms on active sites can enhance the N_2_ adsorption rates, providing a new strategic direction for the rational design of TM-based energy-efficient nitrogen fixation catalysts.

## Data Availability

The data presented in this study are available on request from the corresponding author.

## Supplementary Materials

The following supporting information can be downloaded at: https://www.mdpi.com/article/10.3390/ijms23136976/s1. References [51,52,53,54,55] are cited in the supplementary materials.
