# Supplementary material for: Experimental and Theoretical Study of N2 Adsorption on Hydrogenated Y2C4H− and Dehydrogenated Y2C4− Cluster Anions at Room Temperature
Source: Int J Mol Sci. 2022 Jun 23;23(13):6976. doi: 10.3390/ijms23136976 (PMC9266966; doi:10.3390/ijms23136976)
Supplement: Supplementary file 1 [file ijms-23-06976-s001.zip › Supporting Information-ijms-1739166 final.pdf]

Supplementary Information for

Experimental and Theoretical Study of N_2 Adsorption on Hydrogenated $Y_2C_4H^-$ and Dehydrogenated $Y_2C_4^-$ Cluster Anions at Room Temperature

Min Gao, Yong-Qi Ding and Jia-Bi Ma *

Key Laboratory of Cluster Science of Ministry of Education, Beijing Key Laboratory of Photoelectronic/Electrophotonic Conversion Materials, School of Chemistry and Chemical Engineering, Beijing Institute of Technology, Beijing 102488, China

* Correspondence: majiabi@bit.edu.cn

Contents:

1. Additional time-of-flight (TOF) mass spectra. (Pages S2, S3)
2. Additional density functional theory results. (Pages S5-S16)
3. References. (Page S17)

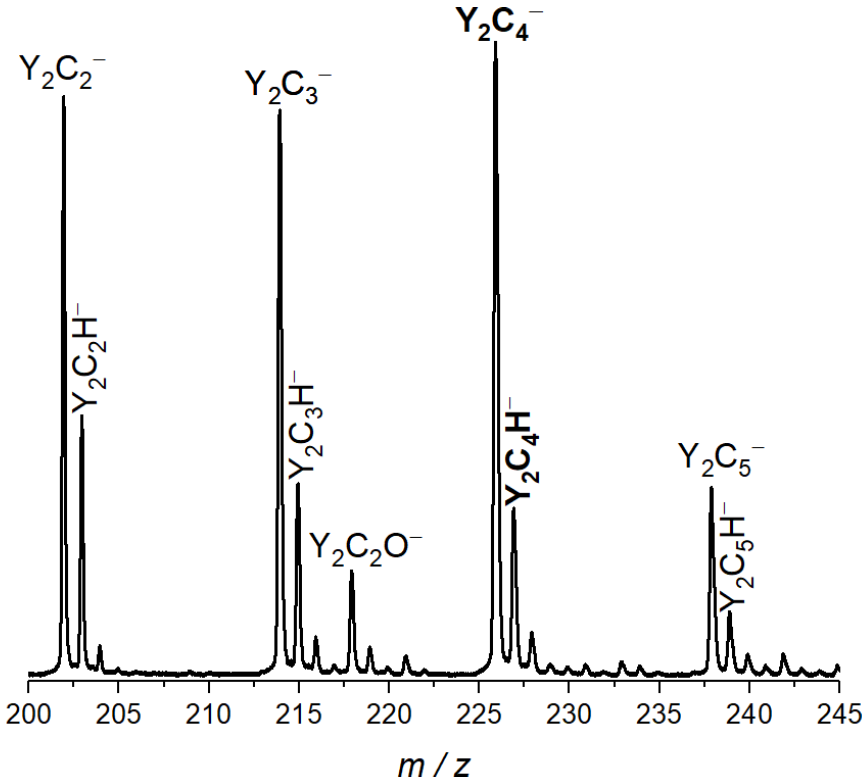

Figure S1. The mass spectra for the generation of $Y_2C_4H_{0,1}^-$.

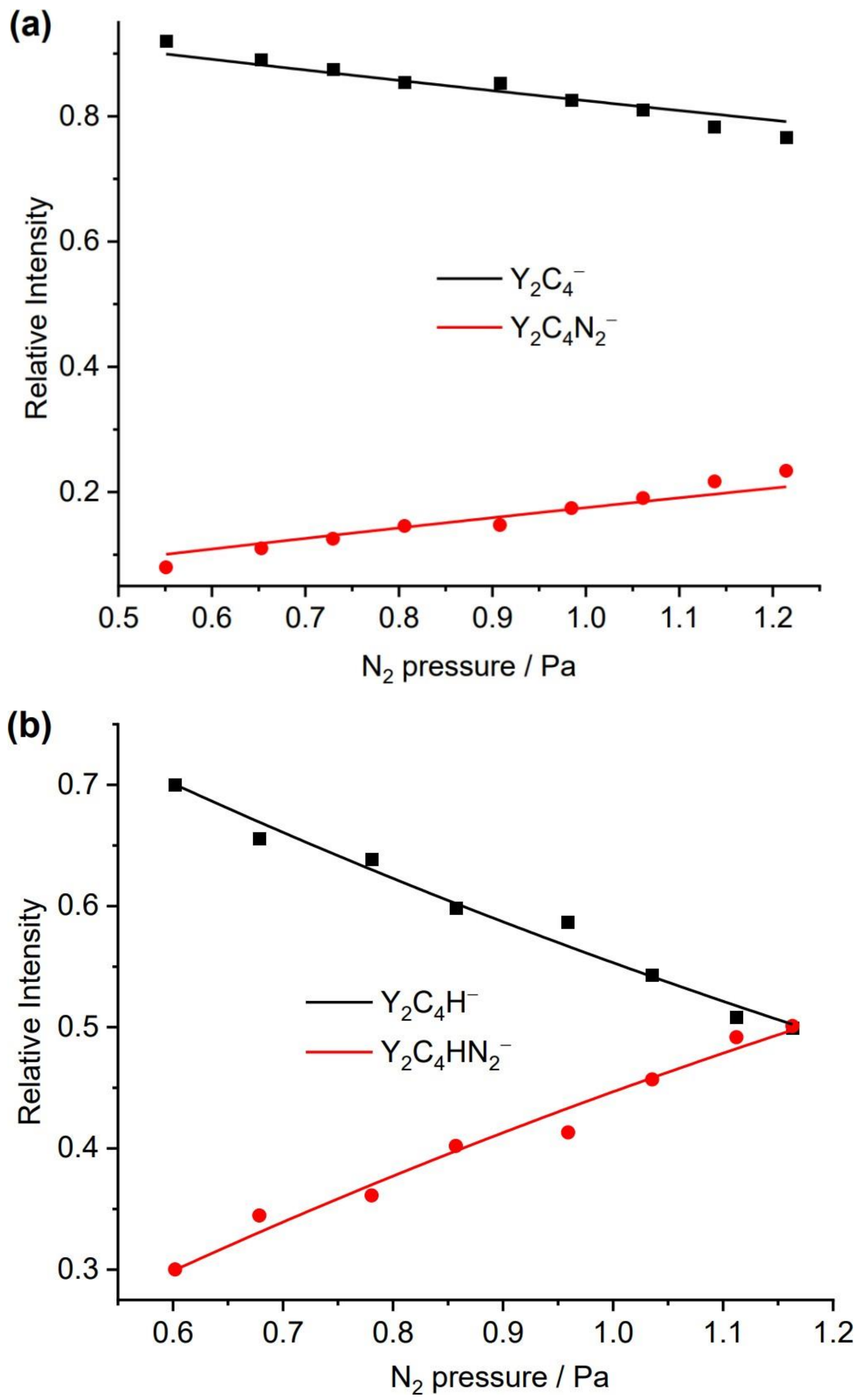

Figure S2. Variations of the relative ion intensities with respect to the N_2 pressures in the reactions of (a) $Y_2C_4^-$ and N_2 for 6 ms, as well as (b) $Y_2C_4H^-$ and N_2 for 14 ms, respectively. The solid lines are fitted to the experimental data points by using the equations derived with the approximation of the pseudo−first−order reaction mechanism.

The Rice–Ramsperger–Kassel–Marcus theory (RRKM)[33] was used to calculate the rate constant of traversing transition states from intermediates. For these calculations, the energy (E) of the reaction intermediate and the energy barrier ($E^{‡}$) for each step were needed. The reaction intermediate possesses the vibrational energies (E_{vib}) of $Y_2C_4H_{0,1}^-$ and N_2, the center of mass kinetic energy (E_k), and the binding energy (E_b) which is the energy difference between the separated reactants ($Y_2C_4H_{0,1}^-$ + N_2) and the reaction complexes. The values of E_{vib} and E_b were taken from the DFT calculations and $E_k = \mu v^2/2$, in which μ is the reduced mass and v is the velocity. The densities and the numbers of states required for RRKM calculations were obtained by the direct count method[51] with the DFT calculated vibrational frequencies under the approximation of harmonic vibrations. According to the DFT calculated energies, the rates of internal conversion ($k_{conversion}$) for processes of **I2** → **TS2** in Reaction (a) and **I4** → **TS4** in Reaction (b) are 2.65×10^{10} s^{-1} and 8.49×10^{11} s^{-1}, respectively.

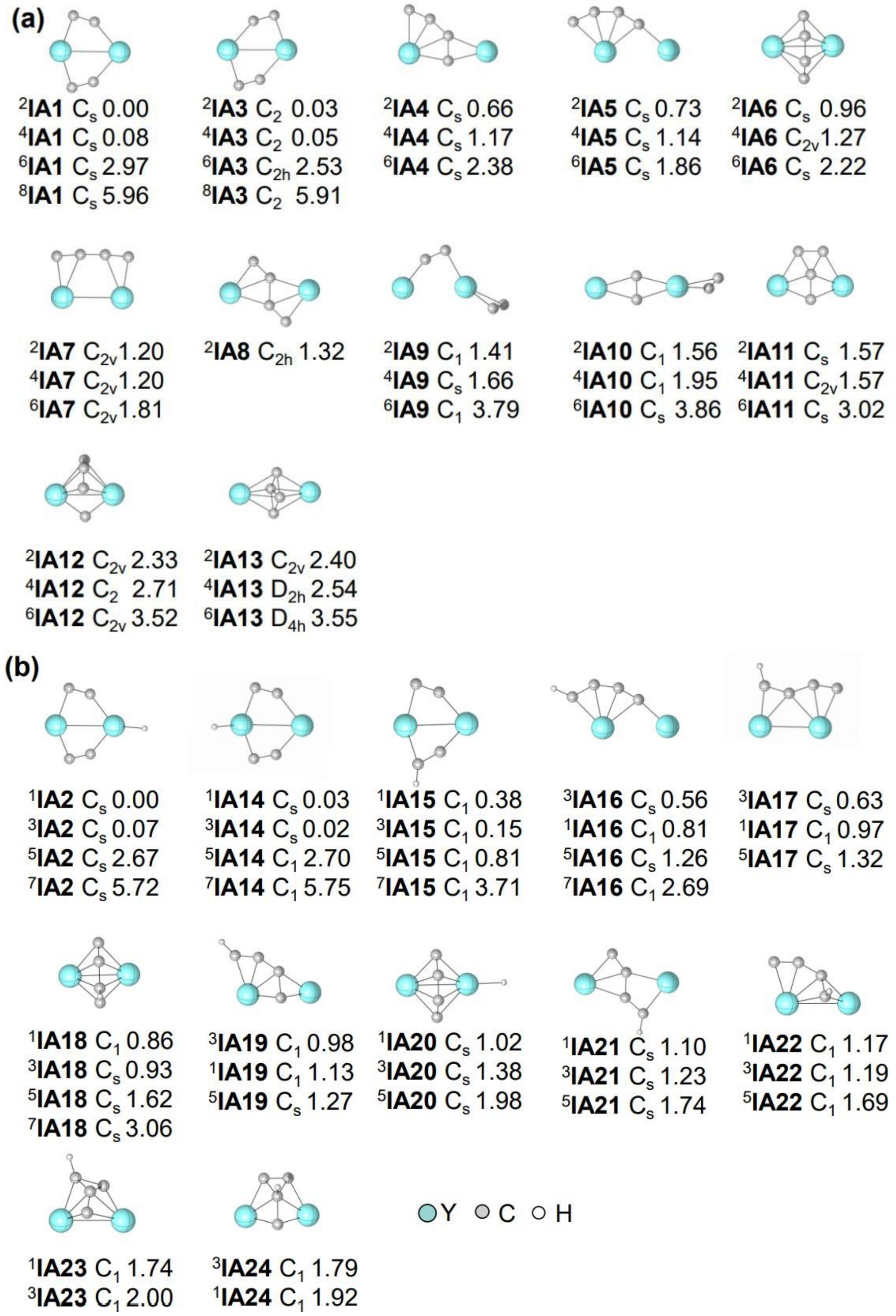

Figure S3. DFT−calculated structures and relative energies of $Y_2C_4H_{0,1}^-$. The point group and electronic state are given under each structure. Superscripts indicate the spin multiplicities. The zero−point vibration corrected energies (ΔH_{0K} in eV) of each structure are given.

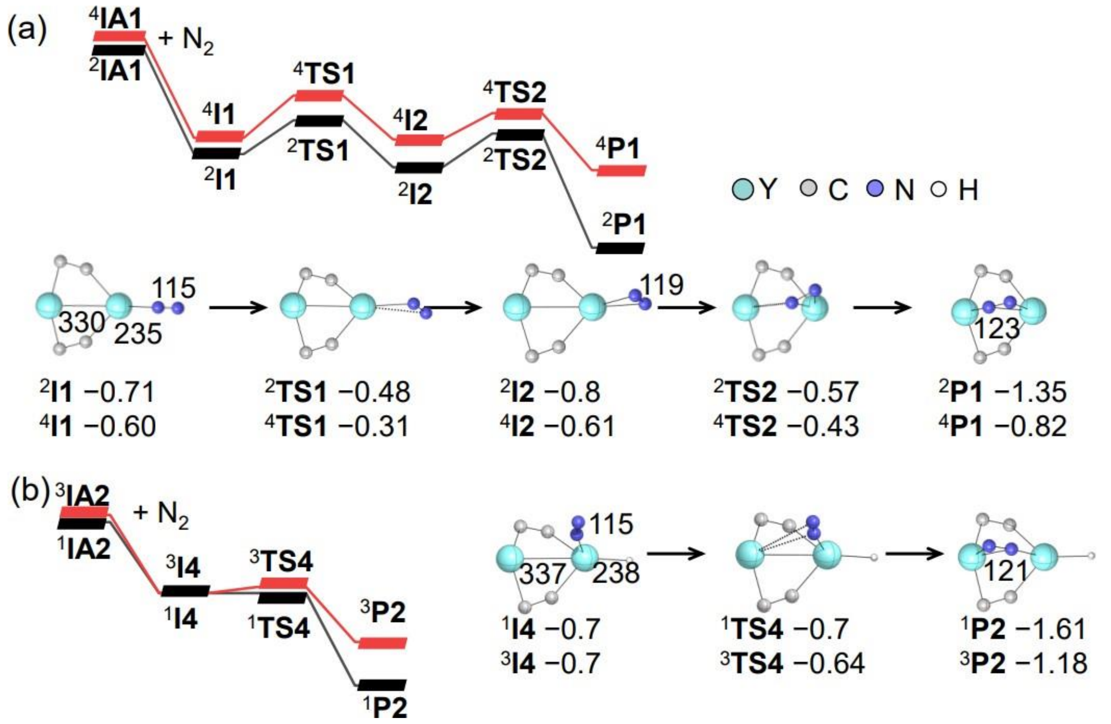

Figure S4. BPW91−D3−calculated potential energy surfaces for the reactions of (a) $Y_2C_4^-$ and (b) $Y_2C_4H^-$ with N_2. The spin multiplicities of (a) are doublet and quartet. The spin multiplicities of (b) are singlet and triplet. Single−point energy calculations by DFT were performed to determine the relative energies (in eV) of the intermediates, transition states, and products with respect to the separate reactants.

Table S1. Enthalpy and Gibbs free energies along with electronic and zero−point correction energies. Energies in eV are given.

structure	Enthalpy (eV)	Gibbs free energies (eV)	EE + Zero−point Energy(eV)
^{2}**IA1**+N_2	0	0	0
^{2}**I1**	−0.71	−0.34	−0.71
^{2}**TS1**	−0.49	−0.12	−0.48
^{2}**I2**	−0.81	−0.42	−0.8
^{2}**TS2**	−0.60	−0.13	−0.57
^{2}**P1**	−1.39	−0.89	−1.35
^{4}**IA1**+N_2	0.08	0.07	0.08
^{4}**I1**	−0.60	−0.27	−0.6
^{4}**TS1**	−0.33	0.03	−0.31
^{4}**I2**	−0.62	−0.26	−0.61
^{4}**TS2**	−0.46	−0.01	−0.43
^{4}**P1**	−0.85	−0.40	−0.82

structure	Enthalpy (eV)	Gibbs free energies (eV)	EE + Zero−point Energy(eV)
^{1}**IA2**+N2	0	0	0
^{1}**I4**	−0.72	−0.31	−0.70
^{1}**TS4**	−0.74	−0.26	−0.70
^{1}**P2**	−1.66	−1.14	−1.61
^{3}**IA1**+N_2	0.07	0.049	0.07
^{3}**I4**	−0.70	−0.38	−0.70
^{3}**TS4**	−0.66	−0.27	−0.64
^{3}**P2**	−1.22	−0.74	−1.18
^{1}**I6**	−0.46	−0.09	−0.45
^{1}**TS6**	−0.26	0.15	−0.24
^{1}**I7**	−0.59	−0.19	−0.58
^{1}**TS7**	−0.39	0.055	−0.37

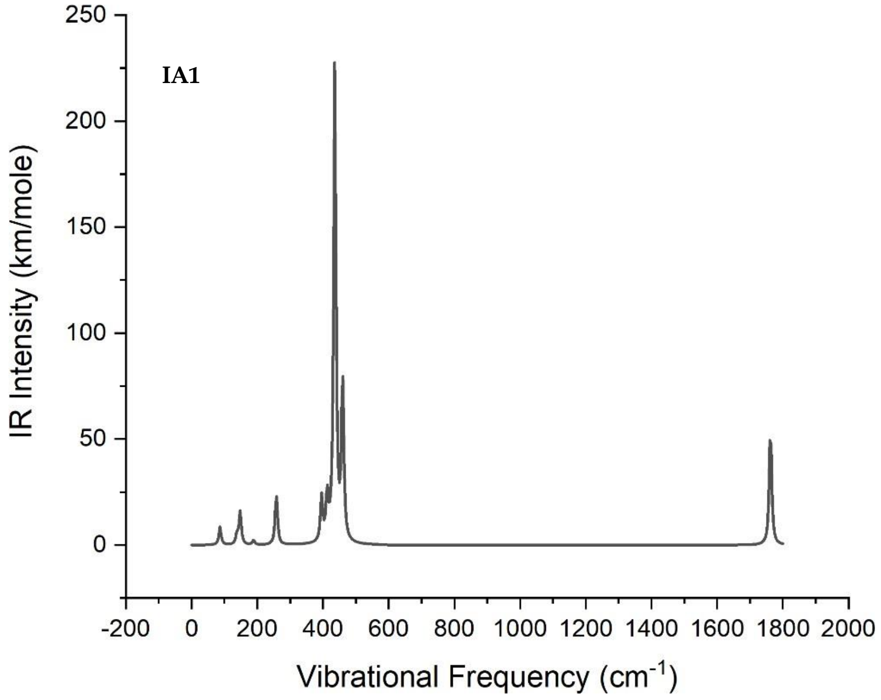

IA1
IR Intensity (km/mole)
Vibrational Frequency (cm-1)

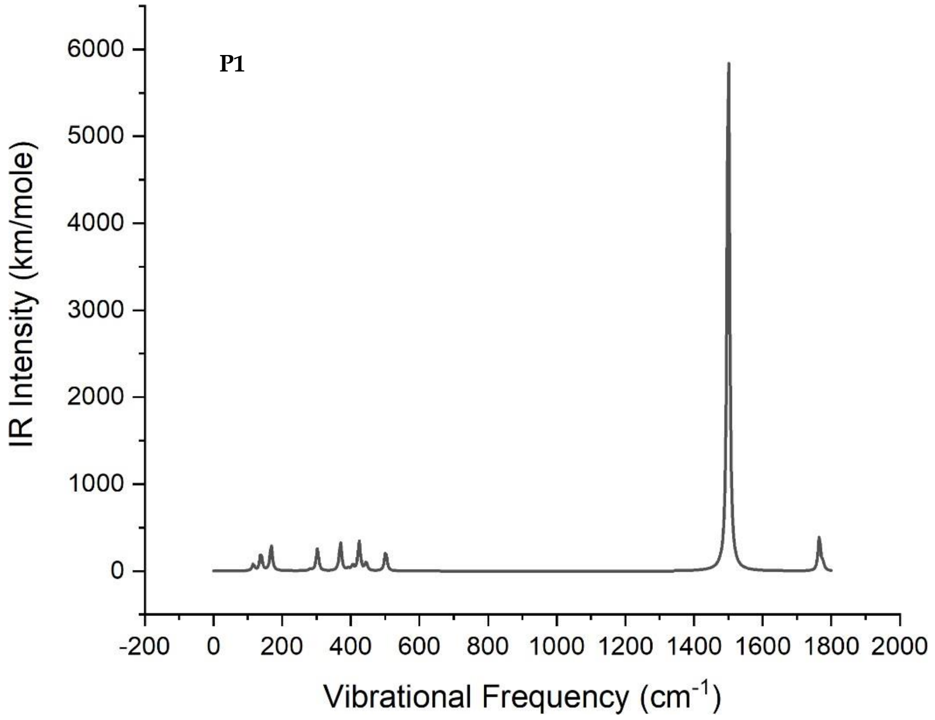

P1
IR Intensity (km/mole)
Vibrational Frequency (cm-1)

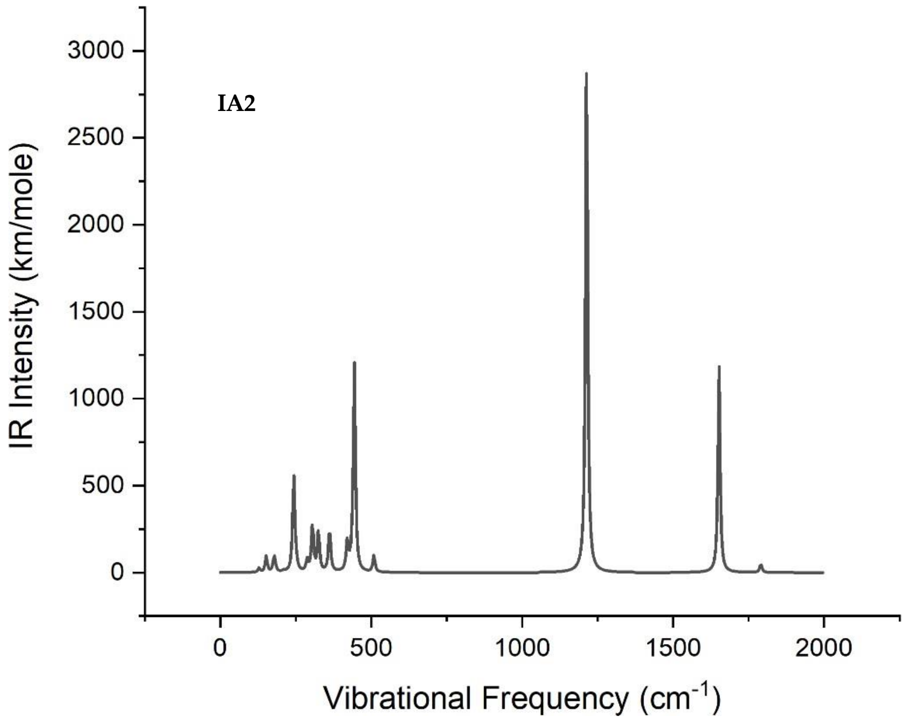

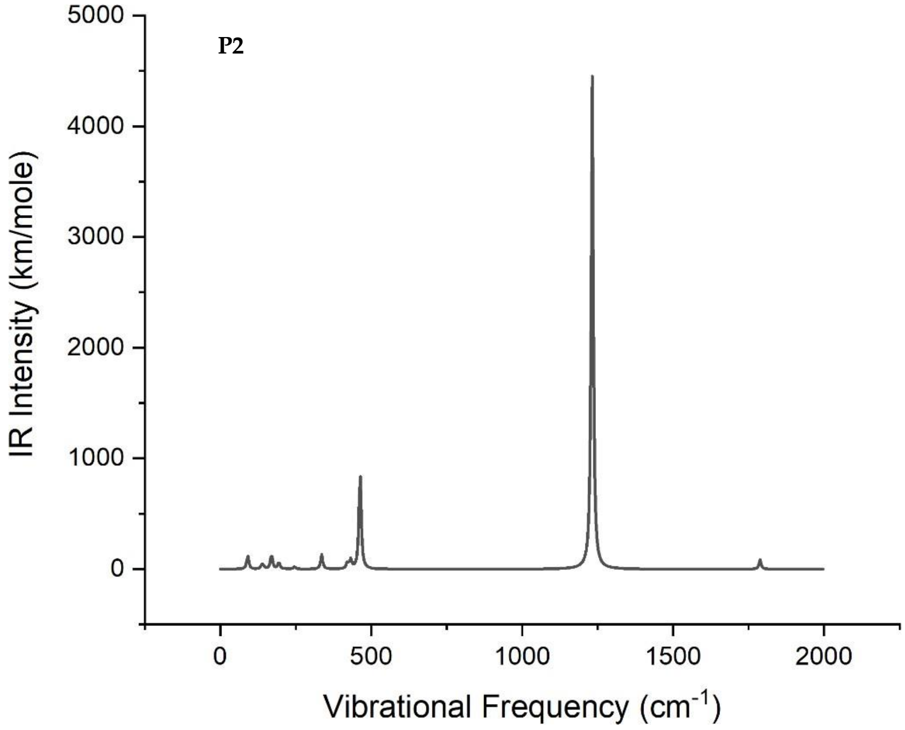

Figure S5. Density functional theory calculated infrared spectra of **IA1, IA2, P1** and **P2.**

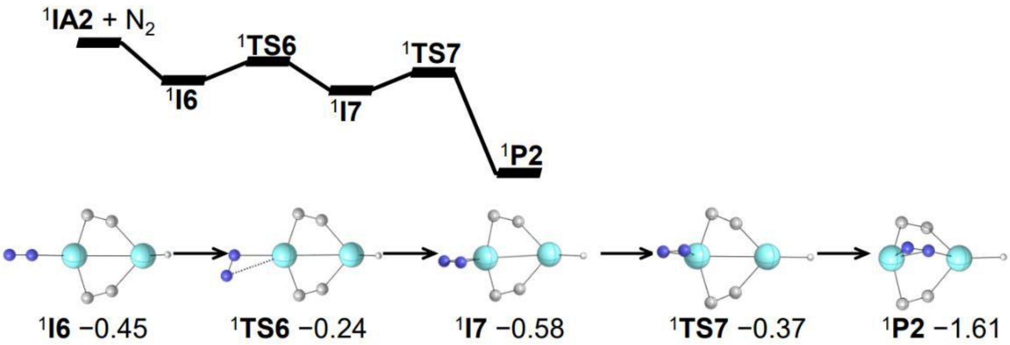

Figure S6. BPW91−D3−calculated potential energy surface for the reaction of $Y_2C_4H^-$ with N_2. The zero−point vibration−corrected energies (ΔH_{0K} in eV) of the reaction intermediates, transition states, and products with respect to the separated reactants are given. The superscripts indicate the spin multiplicities.

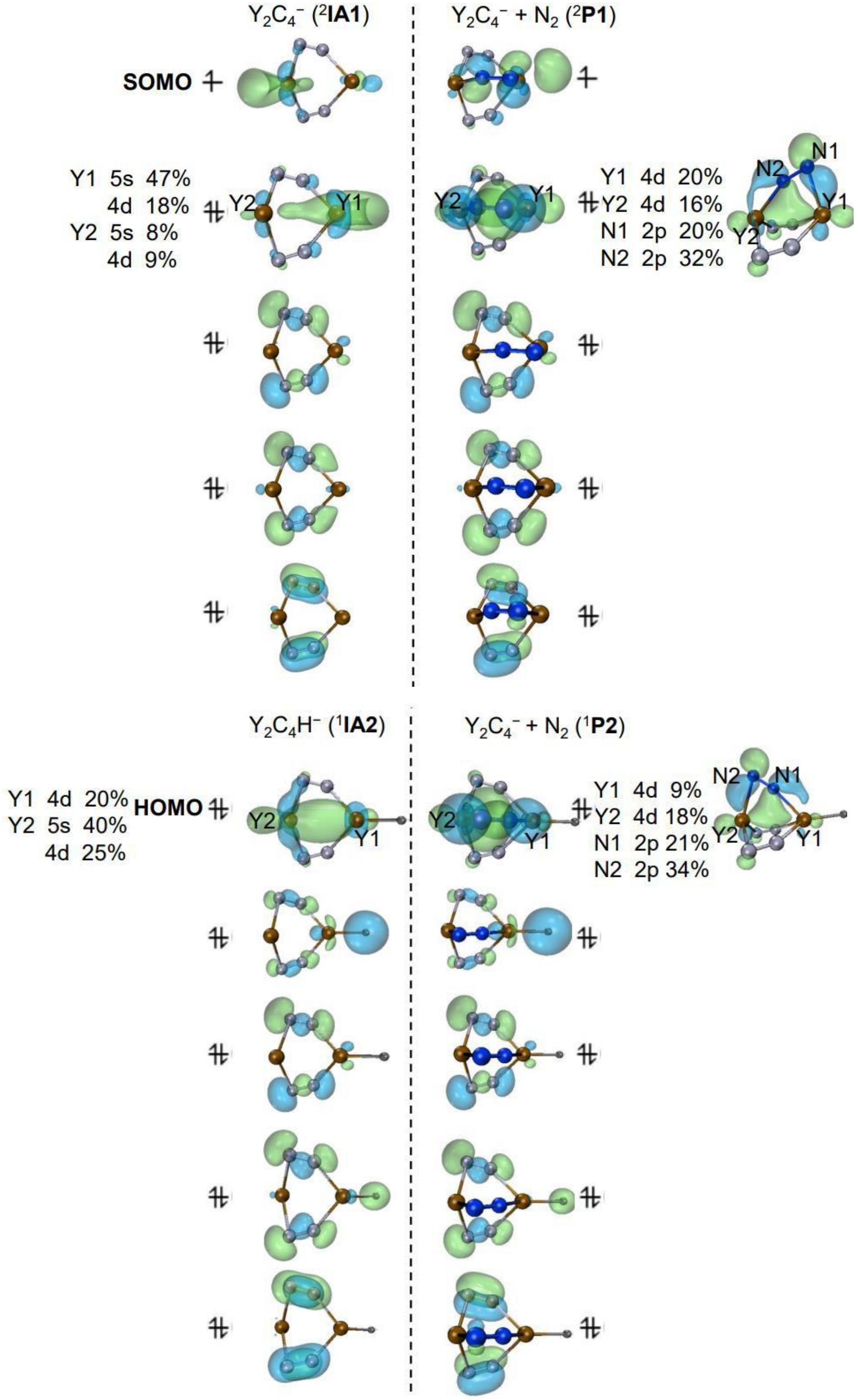

Figure S7. Schematic molecular orbital diagrams for (a) the $Y_2C_4^-$ (^{2}**P1**) and (b) $Y_2C_4H^-$ (^{1}**P2**) in the pathways shown in Figure 2, respectively

Table S2. Charge details about the species in reaction pathway are given.

structure	^{2}**IA1**	^{2}**I1**	^{2}**TS1**	^{2}**I2**	^{2}**TS2**	^{2}**P1**
C1	−0.53850	−0.55204	−0.53423	−0.56114	−0.57873	−0.55014
C2	−0.81981	−0.77152	−0.80839	−0.80420	−0.76200	−0.74067
C3	−0.81981	−0.77152	−0.80839	−0.80412	−0.75927	−0.73551
C4	−0.53850	−0.55204	−0.53423	−0.56102	−0.57613	−0.56829
Y1	0.78572	1.14426	1.30755	1.52441	1.11711	1.16300
Y2	0.93089	0.97876	0.91770	0.94891	1.22379	1.43432
N1		−0.36811	−0.39057	−0.36486	−0.31526	−0.56119
N2		−0.10779	−0.14944	−0.37797	−0.34951	−0.44153

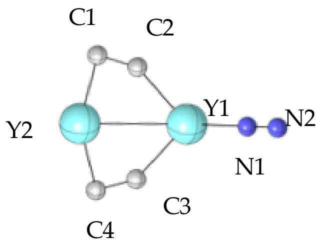

structure	[1]**IA2**	[1]**I4**	[1]**TS4**	[1]**P2**
C1	−0.54547	−0.55496	−0.56108	−0.54738
C2	−0.78534	−0.74573	−0.74534	−0.73381
C3	−0.54547	−0.55496	−0.56108	−0.54738
C4	−0.78534	−0.74573	−0.74534	−0.73381
H	−0.61720	−0.60910	−0.61434	−0.63527
Y1	1.47701	1.49931	1.49066	1.56622
Y2	0.80183	1.11106	1.18165	1.49724
N1		−0.29377	−0.31855	−0.48671
N2		−0.10613	−0.12658	−0.37911

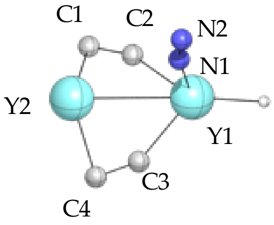

Table S3. DFT−calculated and experimental bond dissociation energies. Energies in eV are given.

Methods \ EXP.		Y–C	C–C	Y–N	N≡N	A.D.[1]
		4.332 ±0.653	6.37 ±0.12	4.944 ±0.653	9.79 ±0.001	
References		52	53	54	55	
Hybrid Functionals	B1B95	3.08	6.01	3.88	9.44	0.76
	B1LYP	3.08	5.77	3.56	9.33	0.92
	B3LYP	3.30	5.98	3.87	9.60	0.67
	B3P86	3.68	6.24	4.25	9.78	0.37
	B3PW91	3.61	6.08	4.02	9.43	0.58
	M05	3.15	6.11	4.10	9.36	0.68
	M052X	2.85	5.82	3.43	9.46	0.97
	PBE1PBE	3.60	6.10	3.91	9.42	0.60
	X3LYP	3.28	5.97	3.80	9.58	0.70
	M06	3.10	6.04	3.98	9.27	0.76
	M062X	2.10	5.91	3.36	9.44	1.16
	BH&HLYP	2.83	5.23	2.78	8.63	1.49
	BMK	3.05	5.77	3.38	9.49	0.94
Pure Functionals	**BPW91**	**3.88**	**6.50**	**4.66**	**9.95**	**0.26**
	BLYP	3.50	6.35	4.46	10.09	0.41
	BP86	3.87	6.61	4.76	10.25	0.34
	BPBE	3.90	6.55	4.68	9.95	0.26
	M06L	3.87	6.34	4.44	9.41	0.35
	PBE	3.95	6.72	4.77	10.24	0.34
	TPSS	3.76	6.20	4.44	9.53	0.38

$A.D. = \frac{\sum (x_i - x_{exp})}{4}$, x_i is the DFT calculated bond dissociation energy and x_{exp} is the experimental value.

The Cartesian coordinates of all structures on display (M is denoted as spin state)

Coordinate (Å) [2]**IA1**

**C**	0.44963800	0.24866100	1.70426000
**C**	0.44963800	-0.98023100	-2.05139700
**C**	0.44963800	0.24866100	-1.70426000
**C**	0.44963800	-0.98023100	2.05139700
**Y**	0.12473300	1.76076400	0.00000000
**Y**	-0.40143300	-1.53566500	0.00000000

Coordinate (Å) [2]**I1**

**C**	0.73615600	-0.22583700	1.71007700
**C**	0.73615600	-1.45278800	-2.05668500
**C**	0.73615600	-0.22583700	-1.71007700
**C**	0.73615600	-1.45278800	2.05668500
**Y**	0.28696100	1.28355800	0.00000000
**Y**	-0.12105200	-1.99255300	0.00000000
**N**	-1.33006000	2.99250500	0.00000000
**N**	-2.11825300	3.83525600	0.00000000

Coordinate (Å) [2]**I2**

**C**	0.43698000	-0.29009300	1.70004100
**C**	0.43698000	-1.51736700	-2.05091800
**C**	0.43698000	-0.29009300	-1.70004100
**C**	0.43698000	-1.51736700	2.05091800
**Y**	0.26863800	1.29646000	0.00000000
**Y**	-0.45022400	-2.01269400	0.00000000
**N**	-0.82706100	3.42221700	0.00000000
**N**	0.34053500	3.66673300	0.00000000

Coordinate (Å) [2]**P1**

**C**	0.07040900	-1.32989900	-1.35934900
**C**	-1.27447600	2.16980000	-0.29842500
**C**	-0.04525700	1.83632000	-0.36487400
**C**	-1.16654300	-1.63352000	-1.45159800
**Y**	1.58946000	0.14608300	-0.30012000
**Y**	-1.50868200	-0.08595000	0.23166400
**N**	0.22688900	-0.50134700	1.53123300
**N**	1.39381000	-0.72742900	1.82808700

Coordinate (Å) [1]**IA2**

C	-0.52011100	-1.01388400	2.03408200
C	-0.52011100	0.22236500	1.72472500

C	-0.52011100	-1.01388400	-2.03408200
C	-0.52011100	0.22236500	-1.72472500
H	0.71531100	3.65186500	0.00000000
Y	0.37518900	-1.56489300	0.00000000
Y	-0.07346200	1.71479900	0.00000000

Coordinate (Å) [1]**I4**

C	0.81969400	1.26130100	2.03097100
C	0.81969400	0.04135800	1.65912800
C	0.81969400	1.26130100	-2.03097100
C	0.81969400	0.04135800	-1.65912800
H	1.05478100	-3.56562300	0.00000000
Y	-0.06551500	1.76515900	0.00000000
Y	0.43284300	-1.57209900	0.00000000
N	-1.93439500	-1.33683900	0.00000000
N	-3.07320800	-1.46253600	0.00000000

Coordinate (Å) [1]**P2**

C	-0.88696700	-1.16421700	1.98863400
C	-0.88696700	0.06868300	1.66557900
C	-0.88696700	-1.16421700	-1.98863400
C	-0.88696700	0.06868300	-1.66557900
H	-0.61728800	3.78493400	0.00000000
Y	0.28646200	-1.47093800	0.00000000
Y	-0.42634700	1.68343200	0.00000000
N	1.52500300	0.50182600	0.00000000
N	2.38357200	-0.34836800	0.00000000

Coordinate (Å) [1]**I7**

**C**	-0.30436600	2.00451500	-0.95061600
**C**	0.74407800	1.69622100	-0.28789900
**C**	-0.30393000	-2.00473800	-0.95061400
**C**	0.74414900	-1.69612600	-0.28746300
**H**	3.20578300	0.00021300	2.01245800
**Y**	-1.20933700	-0.00009300	-0.12285000
**Y**	2.21175000	0.00006400	0.20699000
**N**	-3.59138200	0.00001700	0.12465000
**N**	-3.20568800	0.00022400	1.24186800

References

33. Steinfeld, J.I.; Francisco, J.S.; Hase, W.L. *Chemical Kinetics and Dynamics*; Prentice-Hall: Hobboken, NJ, USA, 1999; p. 231.
51. Beyer, T.; Swinehart, D.F. Algorithm 448: Number of Multiply-Restricted Partitions. *Commun. ACM* **1973**, *16*, 379.
52. Simoes, J.A.M.; Beauchamp, J. L. Transition metal-hydrogen and metal-carbon bond strengths: The keys to catalysis. *Chem. Rev.* **1990**, *90*, 629–688.
53. NIST Chemistry Webbook; Mallard, W.G., Ed.; August 1996. Available online: http://webbook.nist.gov (accessed on 20 January 2022).
54. Gurvich, L.V.; Karachevtsev, G.V. *Bond Energies of Chemical Bonds, Ionization Potentials and Electron Affinities*; Nauka: Moscow, Russia, 1974.
55. Tang, X.N.; Hou, Y.; Ng, C.Y.; Ruscic, B. Pulsed field-ionization photoelectronphotoion coincidence study of the process $N_2 + h\nu \rightarrow N^+ + N + e^-$: Bond dissociation energies of N_2 and N_2^+. *J. Chem. Phys.* **2011**, *123*, 074330.
